# Influence of Dietary Zinc, Copper, and Manganese on the Intestinal Health of Broilers Under *Eimeria* Challenge

**DOI:** 10.3389/fvets.2020.00013

**Published:** 2020-01-28

**Authors:** Cristiano Bortoluzzi, Bruno Serpa Vieira, Todd Jay Applegate

**Affiliations:** ^1^Department of Poultry Science, Texas A&M Agrilife Research, College Station, TX, United States; ^2^Federal Institute of Education, Science and Technology of Mato Grosso, Cuiabá, Brazil; ^3^Department of Poultry Science, University of Georgia, Athens, GA, United States

**Keywords:** broilers, coccidiosis, copper, manganese, zinc

## Abstract

The incidence of enteric infections in broiler chickens may increase worldwide due to mounting pressure to limit the use of sub-therapeutic antibiotics and ionophores for coccidia suppression/prevention in the diets of broilers. For this reason, we need expand our knowledge on the role that micro-minerals have in modulating the intestinal physiology, immunology, and microbiology of broiler chickens. There are issues associated with the use of high doses of some micro-minerals in the diets of animals, such as environmental contamination, bacterial resistance, and gizzard erosion. Therefore, there is a need to maximize the absorption of these minerals by the gastrointestinal tract (GIT) of birds when intestinal function may be compromised. Zinc is an essential micromineral required for growth, and influences intestinal development and/or regeneration during and after enteric disease. Two studies were conducted by our lab to determine the effects of Zn source in broilers under coccidia and *Clostridium perfringens* challenge. In the first study, Zn proteinate had beneficial effects on the performance of chickens challenged with coccidia plus *C. perfringens* by enhancing intestinal integrity and partially attenuating the inflammatory response. In the second study, Zn proteinate lowered the expression of pro-inflammatory cytokines and modulated the ileal microbiota. Additionally, the poultry industry has used prophylactic concentrations of dietary Cu for its ability to improve feed conversion for a long time. Copper absorption occurs mainly in the duodenum of chickens and, therefore, injuries to the intestinal epithelium of duodenum would impair Cu absorption and decrease its tissue concentration. Although there is a lack of studies relating Mn supplementation and its different sources on the immune response against coccidiosis in poultry, it is likely that Mn is beneficial during enteric challenges due to its role in the production of mucopolysaccharides. Therefore, the proper evaluation of the role of minerals on mitigating the negative impact of coccidiosis in broilers must consider their properties in modulating the physiology, immunology, and the intestinal microbiota of the host during health and disease situation events.

## Introduction

Coccidiosis is an enteric disease caused by protozoa of the genus *Eimeria* that affects several animal species, including poultry. *Eimeria* multiplication in the intestinal epithelium leads to enterocyte destruction and severe tissue damage, which negatively affects poultry health and welfare status (1). Although difficult to predict, researchers have estimated that the loss in productivity induced by clinical and subclinical coccidiosis in poultry, together with the expenses in prevention and treatment, cost to the industry an excess of US$ 2 billion annually (2, 3).

Besides increasing the susceptibility to NE, coccidiosis also leads to changes in the overall structure of the intestinal microbiota (4, 5). Wu et al. (5) demonstrated a reduction in the cecal microbial diversity following *Eimeria* infection, and reduction of many members of the family *Ruminococcaceae*. Yet, *Eimeria* infection caused changes in short-chain fatty acids (SCFA) produced in the ceca of chickens (4).

Macro and microminerals have long been known to directly or indirectly influence poultry gastro-intestinal health, metabolism and growth performance. Trace minerals like Fe and Cu, for instance, can act as pro-oxidants, reducing the stability of vitamins and enzymes and promoting oxidation of lipids (6). Finely ground CuSO_4_ results in increased rates of fat oxidation in the feed, as compared to coarsely ground CuSO_4_ (7). Zinc and Cu are acknowledged to be important modulators of the endogenous mechanisms of defense against infections, inflammations and oxidative stress (8–13). Also, the antimicrobial effect of Cu against *Escherichia coli* and other pathogens in the intestine of broilers has already been demonstrated (14, 15). However, there is a clear evidence that bacteria also develop resistance against minerals specially against Zn and Cu that are used in the feed due to their antimicrobial properties (16).

Generally speaking, the chemical form in which minerals are presented in the diet, together with several other factors such as the total mineral concentration in the feed, particle size, feed processing, strain and age of the animal, and the potential of the mineral to interact with other dietary components have been proved to influence mineral availability and, as a consequence, their effects on the physiology of animals (7). Additionally, the distribution of minerals within the body of the animal may change during enteric infections (17–21), which could be the consequence of shifts in the mineral metabolism during the course of the disease and/or simply the result of different rates of mineral absorption induced by the damage of intestinal mucosa and disruption of the regular intestinal integrity. Despite the reason, it is reasonable to argue that when the absorptive capacity of the intestine is impaired due to enteric infections such as coccidiosis, sources of minerals of greater availability may be needed.

Trace minerals supplementation is not novelty to poultry nutrition, but producers are now feeding them at higher levels to increase birds' intestinal health. Several products have been launched by the feed industry with that objective, most of them formulated with Zn, Cu, and/or Mn, also the most studied trace minerals in poultry (22, 23). Although we know that some trace minerals have antimicrobial effects, there is still a lack of knowledge whether these minerals also have a direct effect against *Eimeria*, or just indirectly through their antimicrobial and immunomodulatory effect. With that in mind, and considering that the use of antibiotics or other traditional molecules to prevent enteric diseases in poultry has been constantly put into check by consumers, the objective of this study was to review the literature regarding the role of microminerals, particularly Zn, Cu, and Mn, in mitigating the negative impact of coccidiosis in broilers.

## Minerals Interactions Within the GIT

When minerals from inorganic sources are fed and reach the upper parts of the gastro-intestinal tract (GIT) they tend to dissociate due to the low pH and be absorbed. In the lower GIT, however, the higher luminal pH increases the interaction between mineral cations and other dietary components (9), leading to the formation of insoluble complexes with much lower availability to animals. Not by chance, higher rates of mineral absorption in broilers have been described in the duodenum rather than in more distal segments of the intestine (24).

The widest known and studied example of these insoluble complexes is probably phytate, originated from phytic acid and its 12 reactive sites (6 strongly acidic, 2 weakly acidic, and 4 very weakly acidic) (25). Macro and micromineral cations, particularly Ca, Zn, and Cu, bind readily to phytic acid as pH increases above 4, forming insoluble complexes that not only decrease the availability of minerals, but also the hydrolytic function of phytases (25–27).

Organic or chelated form of microminerals may have higher bioavailability than inorganic sources, mainly because of their different route of absorption and lower interaction with other dietary constituents. Chelated minerals are metallic ions bound to organic substances, such as amino acids, peptides, or polysaccharides that create a stable and soluble molecule with high bioavailability (28). Chelated minerals show superior absorption than inorganic forms because usually the mineral is absorbed through the path of the organic ligand that the ion is bound, avoiding its interaction with other molecules. The mechanism by which the ligand improves the use of the mineral depends upon the capacity of the ligand to bind to the mineral and its capacity to compete with other ligands, creating soluble complexes with the mineral (29).

The mineral cation-phytate complex that forms within the GIT theoretically explains much of the differences in efficacy that it is observed between phytase sources with differing pH profiles, with much of the improvement with today's generation of phytases occurring because the profiles favor hydrolysis at more acidic pH where the cation-phytin complexes are more soluble (26, 30). However, the consistency of results observed *in vitro* with mineral cations is not always translated into predictable and quantifiable results across ages and mineral sources within and between research labs.

For example, across 5 studies with Cu in broiler chicks (15, 31–33), Cu did not reduce the apparent ileal P or phytate P (PP) hydrolysis when included up to 188 mg/kg of diet. However, when the dietary Cu concentration was increased to 250 mg/kg, it reduced apparent ileal P digestibility by 0.03 to 0.05 percentage—units of the diet. There appears to be differences between Cu sources. For example, as observed by Banks et al. (32), supplementation of 250 mg/kg of Cu chloride or Cu lysinate improved apparent P retention when compared to Cu citrate or Cu sulfate.

Similarly, a threshold of dietary concentrations of minerals needed to affect *in vivo* P digestibility or PP hydrolysis may exist. For example, results from a turkey poult study where up to 161 mg/kg of dietary Zn was fed showed no differences in apparent ileal P digestibility or PP hydrolysis (Applegate et al., unpublished results). Thus, the concentration of dietary Cu and Zn needed to have a consistent negative impact on P digestibility or PP hydrolysis may be above concentrations used in practical poultry diets. In addition to the effects on P digestibility, Rochell et al. (34) observed that Cu supplementation increased apparent ileal digestibility of total amino acids in birds fed low amino acid concentration but decreased it in birds fed high amino acid concentration. Additionally, these authors observed that the organic matter digestibility was reduced in *Eimeria* infected birds and supplemented with Cu.

Calcium, while on one hand forms weaker complexes with individual phytate molecules, may cause more complications due to the high quantities used in the diet (35, 36). These authors demonstrated the effect of Ca on PP digestibility by adding EDTA, a stronger chelator of Ca (vs. phytate), to a broiler diet containing 0.7% Ca. When this diet was fed to broilers, PP digestibility was similar to that seen when a diet devoid of added Ca (0.21% Ca) was fed in a short-term experiment. Thus, in practical broiler diets, presence of Ca inhibits 0.03–0.13% of dietary phytate P hydrolysis (35–37).

### Zinc

Zinc is a micromineral with important function during the recovery of damage caused by enteric diseases (38). The indispensability of Zn in the diets of animals has been recognized for years (39), and traditionally inorganic sources have been used such as oxides and sulfates to supplement the diets of broiler chickens above the NRC (40) recommended concentrations (41). Zinc nutrition has become an active area of research, mainly in broilers. Adequate Zn intake and absorption is essential for many metabolic and biological functions, including growth, reproduction, meat quality, and immune response against pathogens challenge (39).

The distribution of Zn within the animal's body tends to change during periods of infection as shown by different publications over the years (17–21). For example, Bortoluzzi et al. (13) observed that the Zn concentration in the serum decreased and, in the liver, it increased 7 days after challenge with *Eimeria maxima*. Additionally, Zn concentration in the serum slightly increased in birds supplemented with 90 mg/kg of ZnSO4 compared to non-supplemented birds (13). Indeed, severity of growth depression has been observed to be lessened when supplemental Zn was increased to 85–90 mg Zn/kg diet (17, 18, 42). Nevertheless, it has to be taken into consideration that a more accurate description of the distribution of Zn within the body during an intestinal insult (such as that of coccidiosis) is needed.

Studies have shown the impact of Zn on growth performance and antioxidant system (9, 43); immune defense and inflammation (10–13), intestinal permeability (12, 13, 44), and intestinal microbiota (13). Additionally, organic Zn induced higher expression of A20, an anti-inflammatory regulator, downregulated the expression of inflammatory inducers, including NF-kB p65 (10, 11), and promoted MUC2 and IgA production, when compared to its inorganic counterpart (10). Epigenetic mechanisms alter gene expression without changes in DNA sequence and can explain the effects of Zn on the cell. The higher expression of A20 promoted by organic Zn is most likely due to an epigenetic effect by lowering DNA methylation (11). In two recent publications from our lab (7, 8) it has been demonstrated that organic Zn had significant impact in lessening the inflammatory response in the intestine by down-regulating the expression of pro-inflammatory cytokines, such as IL-8 and ING-γ, TLR-2 and iNOS. Additionally, it has been reported that organic Zn reduced the intestinal permeability caused by *Clostridium perfringens* challenge on top of coccidiosis (7), but was not consistent across studies (8).

Looking at the effects of dietary Zn on the intestinal microbial community, Bortoluzzi et al. (8) reported that organic Zn supplementation reduced the amount of *Lactobacillus* in the ileal digesta, and *Coprobacillus* in the cecal content. Furthermore, it seems that high dietary Zn concentrations have no effect (45) or increase the number of enterobacteria in the gut (46), and bacterial metabolites in the small and large intestines were lower in the high dietary Zn group, mainly because of a decrease in propionate concentration, and partially due to lower n-butyrate concentrations. On the other hand, Zn supplementation (120 mg/kg) restored the cecal microbial community of *Salmonella* Typhimurium infected chickens by increasing the number of total bacteria and Lactobacillus, and reducing *Salmonella* colonization (47).

### Copper

The poultry industry has used prophylactic concentrations of dietary Cu for its ability to improve feed conversion for a long time. One of the first reports of Cu supplementation having a growth promoting effect (rather a feed efficiency improvement) was that of Mehring et al. (48). These authors found that the feed efficiency was improved when birds were supplemented with Cu up to the toxic level (500 ppm), suggesting an effect similar to that of the antibiotics. Furthermore, in studies with penicillin and streptomycin, Weeks and Sullivan (49) noted no additional benefit of using Cu in combination with those antibiotics.

Several variables may undergird our knowledge of how source and/or form of Cu may affect the intestinal microbiota. For example, Pang et al. (10) reported that classical plate enumeration of *E. coli* was linearly reduced from media inoculated with ileal digesta from birds fed increasing dietary CuSO_4_ (to 250 mg/kg), but was unaffected by tri-basic copper chloride (TBCC) supplementation up to 250 mg/kg. Conversely, Klasing and Nazipipour (14) observed increased bacteriostatic activity against *E. coli* spiked into ileal content collected from birds fed 150 mg Cu/kg diet from TBCC, but not those from birds fed 150 mg/kg of CuSO_4_. Interestingly, their report related differences observed between Cu sources to that of Cu solubility and extractability from its chemical source. Notably, when CuSO_4_ was fed, duodenal luminal soluble Cu and epithelial metallothionein was increased vs. that from TBCC fed birds. Along the entire small intestine, TBCC resulted in more ethylenebis-hydroxyphenylglycine (EHPG; a strong complexing agent) extractable Cu, thereby suggesting improved bioavailability throughout the length of the GIT.

Both the vertical (proximal to distal) and horizontal (luminal to epithelial associated) distribution of microbiota may then be dependent upon micromineral source and where the mineral is extracted and subsequently absorbed in the luminal/mucosal interface. To this end, Pang et al. (10) noted no change in the number of predominant bacterial populations of either the ileal digesta or intestinal mucosa utilizing a general bacteria-specific PCR primer targeting conserved regions of the V3 region of 16S rRNA. Further, source of Cu (TBCC or CuSO_4_) had no impact on the diversity of bacteria in the intestinal digesta. However, feeding of 187.5 mg/kg Cu from TBCC increased the similarity of bacterial microbiota between birds compared to either control or birds fed 187.5 mg/kg Cu from CuSO_4_. As our technical ability to determine microbiota shifts and what they mean from a host-microbial perspective improves, the poultry science community needs to further elucidate how the chemical form of Cu affect this interface.

Copper absorption occurs mainly in the duodenum of chickens (50). Therefore, it is expected that any injury to the intestinal epithelium of duodenum would impair Cu absorption and decrease its tissue concentration. Nevertheless, Giraldo and Southern (51) found that duodenal coccidiosis resulting from *Eimeria acervulina* infection increases liver Cu concentration. This response may be associated with the usual reduction in the intestinal pH induced by coccidiosis (52), at least during the acute phase of the disease, which would favor the solubility of minerals in the intestinal lumen and improve their absorption rate.

Curiously, blood and liver Cu concentration are positively associated with the plasma ceruloplasmin concentration (53), a multicopper enzyme of the family of acute phase proteins that protects cells against the injuries induced by oxidative stress (53). Therefore, the increment in liver Cu concentration observed during coccidiosis may be an important mechanism of defense associated with lower levels of cellular induced apoptosis. This was already hypothesized by Acetoze et al. (53) when studying the impact of supplementation of Cu and Zn above the requirements of broilers under coccidiosis challenge.

While we know the disassociated cation from some macro and micromineral sources is highly reactive with molecules such as phytate, the poultry industry has included prophylactic concentrations of Cu and Zn in the diet well-beyond what has been indicated to prevent deficiency symptoms. Historically, high concentrations of Cu were originally “touted” as having benefits in prevention of crop mycosis. Indeed, field studies indicate it does have some merit, but reproducibility of induced crop mycosis in experimental conditions has had less than favorable results (54). In fact, addition of up to 250 mg Cu/kg diet results in increased erosion to the lining of the gizzard (55, 56) and results in an “inhibition of normal fermentation” in the ceca of the chick (57). This observation has been confirmed in *in vitro* anaerobic digestion. In particular, volatile fatty acid production can be inhibited considerably due to reductions in microbial activity (58).

### Manganese

A specific biochemical role for Mn in intermediary energy metabolism was confirmed when pyruvate carboxylase was discovered to be a manganese metalloprotein (59, 60). Manganese is required for normal lipid and carbohydrate metabolism through the activity of pyruvate carboxylase. Defects in lipid and carbohydrate metabolism have been reported in Mn-deprived rats and guinea pigs and a diet low in manganese can reduce fat deposition in pigs (61). A second function of Mn has been identified as member of superoxide dismutase (SOD) enzyme. This enzyme is needed for added protection against oxidative stress associated with inflammatory responses to some infections. Manganese deprivation lowers MnSOD activity and increases the peroxidative damage caused by high dietary levels of polyunsaturated fatty acids (PUFA).

Manganese is also needed for the synthesis of mucopolysaccharides through its activation of glycosyltransferase. Impaired glycosyltransferase activity reduces the synthesis of glycosaminoglycan and oligosaccharide side chains in animals deprived of Mn (62). Manganese-deprived chicks have less proteoglycan in the cartilage of the tibial growth plate than Mn replete chicks and the carbohydrate composition of monomers is changed (63). In laying hens, subnormal egg production and poor shell formation may result from impaired mucopolysaccharide synthesis (64). In a work by Ibrahim et al. (65), it was observed that Mn reduced gastric ulceration stimulated by intra-gastric injection of acidified ethanol in rats. Also, the authors showed that Mn increased mucous thickness of the stomach and increased the activity of SOD. However, there is a lack of data on the role of Mn on the production of mucus in the intestine which is considered an important component of the innate immune system of animals.

On the contrary, excessive Mn may accumulate in the mitochondria, disturbing oxidative phosphorylation and increasing the production of reactive oxygen species (ROS) (66). Corroborating with that reasoning, Jankowski et al. (67) found evidences that the complete replacement of Mn oxide by a more available source of the mineral (Mn nanoparticles) in young turkeys' diet increased cellular apoptosis due to oxidative stress. However, the reduction of Mn concentration in the feed decreased the levels of cellular apoptosis irrespective of Mn source.

Manganese is described as a trace mineral associated with better immunity, or to functions that support immunity (67). It has been reported in broiler chickens that on d 6 post-challenge with *E. acervulina* there was a reduction of Mn absorption by 23 and 34% in two consecutive studies, respectively (68); however, there was an increase in Mn absorption compared to uninfected control birds by d 10 after challenge, and then it returned to normal rates as the unchallenged birds. Although there is a lack of studies relating Mn supplementation and its different presentation forms on the immune response against coccidiosis and necrotic enteritis in poultry, one can assume that Mn is beneficial during enteric challenges due to its role in the production of mucopolysaccharides. Burin Junior et al. (69) have shown that birds fed organic Mn had a more efficient response against a *Salmonella* Enteritidis vaccine when compared to birds fed its inorganic counterpart. The absence of effect on the growth performance related to Mn (69) may be attributable to the fact that the basal diet had enough Mn to support growth, and/or the immune stimulus was not strong enough to elucidate a deficiency of Mn. Therefore, it is necessary to understand the dynamics of use of Mn when submitting the birds to a stronger challenge, such as the exposure to *Eimeria* and *C. perfringens*, and how the intestinal immune system could be manipulated.

## Conclusions

Several studies have indicated that the supplementation of microminerals at levels beyond the regular recommendations to broilers may counteract the negative effects of enteric diseases on bird's growth performance and intestinal health. However, care should be taken to minimize environmental contamination, microbial resistance, gizzard erosion, and other concerns when using high concentrations of microminerals in the diets. While some of the paths used by minerals in those responses are already established, most of the time the mechanisms of action are unknown. The high predisposition of minerals to react with each other and with other components of the diet certainly difficult the investigation of their individual effects. Therefore, the proper evaluation of the role of minerals on mitigating the negative impact of coccidiosis in broilers must consider their properties as a whole.

Based on some limitations detected during the literature review process, we suggest for future research that more comprehensive studies describing the changes in body distribution of minerals during coccidiosis and other intestinal diseases should be carried out. Biochemical assays investigating the role of mineral source on cellular metabolism and immune defense against *Eimeria* sp. are desired in order to better explore the benefits of mineral supplementation, particularly Mn, on poultry intestinal health. Additionally, studies focusing on the use of minerals by the *Eimeria* for its replication, and the changes in the animal's requirement of minerals due to coccidiosis must be better investigated.

## Author Contributions

CB and BV did the literature review and wrote the manuscript. TA performed the final review and corrections. All the authors read and approved the last version of the manuscript.

### Conflict of Interest

The authors declare that the research was conducted in the absence of any commercial or financial relationships that could be construed as a potential conflict of interest.
